# Cortical reorganization in an astronaut’s brain after long-duration spaceflight

**DOI:** 10.1007/s00429-015-1054-3

**Published:** 2015-05-12

**Authors:** Athena Demertzi, Angelique Van Ombergen, Elena Tomilovskaya, Ben Jeurissen, Ekaterina Pechenkova, Carol Di Perri, Liudmila Litvinova, Enrico Amico, Alena Rumshiskaya, Ilya Rukavishnikov, Jan Sijbers, Valentin Sinitsyn, Inessa B. Kozlovskaya, Stefan Sunaert, Paul M. Parizel, Paul H. Van de Heyning, Steven Laureys, Floris L. Wuyts

**Affiliations:** Coma Science Group, Cyclotron Research Centre and Neurology Department, University of Liège, Liège, Belgium; Antwerp University Research Centre for Equilibrium and Aerospace (AUREA), Antwerp University Hospital and University of Antwerp, Groenenborgerlaan 171, 2020 Antwerp, Belgium; SSC RF, Institute of Biomedical Problems, Russian Academy of Sciences, Moscow, Russia; iMinds/Vision Lab, University of Antwerp, Antwerp, Belgium; Radiology Department, Federal Center of Treatment and Rehabilitation, Moscow, Russia; Department of Imaging and Pathology, Translational MRI, KU Leuven, Leuven, Belgium; Radiology Department, Antwerp University Hospital and University of Antwerp, Antwerp, Belgium

**Keywords:** Microgravity, Functional MRI, Motor behavior, Cortical reorganization, Vestibular cortex

## Abstract

To date, hampered physiological function after exposure to microgravity has been primarily attributed to deprived peripheral neuro-sensory systems. For the first time, this study elucidates alterations in human brain function after long-duration spaceflight. More specifically, we found significant differences in resting-state functional connectivity between motor cortex and cerebellum, as well as changes within the default mode network. In addition, the cosmonaut showed changes in the supplementary motor areas during a motor imagery task. These results highlight the underlying neural basis for the observed physiological deconditioning due to spaceflight and are relevant for future interplanetary missions and vestibular patients.

**Electronic supplementary material:**

The online version of this article (doi:10.1007/s00429-015-1054-3) contains supplementary material, which is available to authorized users.

Space-travelers face various stressors for their health. These can be physical (e.g., microgravity, ionizing radiation), habitability-related (e.g., noise, vibration), or psychological (e.g., isolation, confinement) (Kanas and Manzey 2008). Although humans mostly adapt to new surroundings, it is not infrequent that some changes are maladaptive with detrimental effects on different physiological systems. To date, the somatic (mal)adaptations have been extensively studied (for a review, see Buckey 2006). However, to our knowledge, no MRI-based neuroimaging study has yet been performed to assess neuronal function in space-travelers. This study aimed at characterizing the impact of long-duration spaceflight on brain function in a single cosmonaut, measured by functional MRI.

A 44-year-old male cosmonaut had his first long-duration mission (169 days) to the International Space Station (ISS) in 2014. During spaceflight, the cosmonaut strictly followed the physical and locomotor training in accordance with the Russian countermeasure system. His overall physical performance showed no abnormalities according to the Institute of Biomedical problems (IMBP, Moscow), monitoring the health of the space travelers in the Russian segment of the ISS. On the day of landing, the sensorimotor assessment showed vestibular ataxia, indicating a dysfunctional vestibular system and proprioception. Clinical investigations 3 days postflight revealed continued impairment of motor coordination, similar to earlier reports (Paloski et al. 1993), but disappearance of vertigo. The fMRI protocol was applied twice: 30 days before launch and 9 days after Earth re-entry. During both assessments, the cosmonaut had a 10-min scanning session in a resting condition and a session while executing active mental imagery tasks (i.e., imagining playing tennis, imagining walking around the rooms of his house). These protocols were chosen on the grounds that they can, respectively, identify changes at the whole-brain level (Heine et al. 2012), at the motor system (Monti et al. 2010), while the navigation task was used as a control task where no changes were a priori expected. A group of age- and gender-matched healthy controls (*n* = 7, mean age 37.6 ± 6.46 years) was included in the analysis to account for data variance (see Online Methods for details).

The resting state assessment encompassed (1) a hypothesis-free exploration of changes in the strength of global connectivity pattern as estimated by the intrinsic connectivity contrast (Martuzzi et al. 2011) (voxel-to-voxel connectivity analysis) and (2) a hypothesis-driven estimation of connectivity changes in six brain networks, namely the default mode, the fronto-parietal, the salience, the auditory, the sensorimotor, and the visual network (seed-to-voxel connectivity analysis). With regards to the measure of intrinsic connectivity contrast, it was found that at post-flight there was reduced connectivity in the right insula (Fig. 1a; *x* = 48, *y* = −6, *z* = 4, *z* value = −4.24, *p*_FDR_ < 0.05 at cluster-level) and ventral posterior cingulate cortex (*x* = 6, *y* = −22, *z* = 24, peak voxel *z* value = −3.95, *p*_FDR_ < 0.05 at cluster-level). With regards to the network-level approach, all networks were characterized by their typical spatial pattern across the group of healthy volunteers (SOM, Fig. 1). Network-level functional connectivity changes at post-flight were identified for the default mode network only. Specifically, reduced connectivity was indentified in areas not typically belonging to the network but classically anticorrelating with it, such as in the precentral gyrus (*x* = 34, *y* = −22, *z* = 64, peak voxel *z* value = −3.85) and the postcentral gyrus (*x* = 30, *y* = −22, *z* = 48, peak voxel *z* value = −3.75); seed-by-seed secondary connectivity analysis identified that the connectivity changes resulted from the seed region placed on the left cerebellum (Fig. 1b). No functional connectivity changes were identified in the other networks. The analysis of the active mental imagery tasks showed that the cosmonaut had higher activation of the supplementary motor area (*x* = 9, *y* = −1, *z* = 67, peak voxel *z* value = 3.11, *p*_uncorrected_ < 0.001) post-flight compared to pre-flight for the tennis paradigm. No difference in brain activation was identified for the spatial navigation task as expected.

**Fig. 1:**
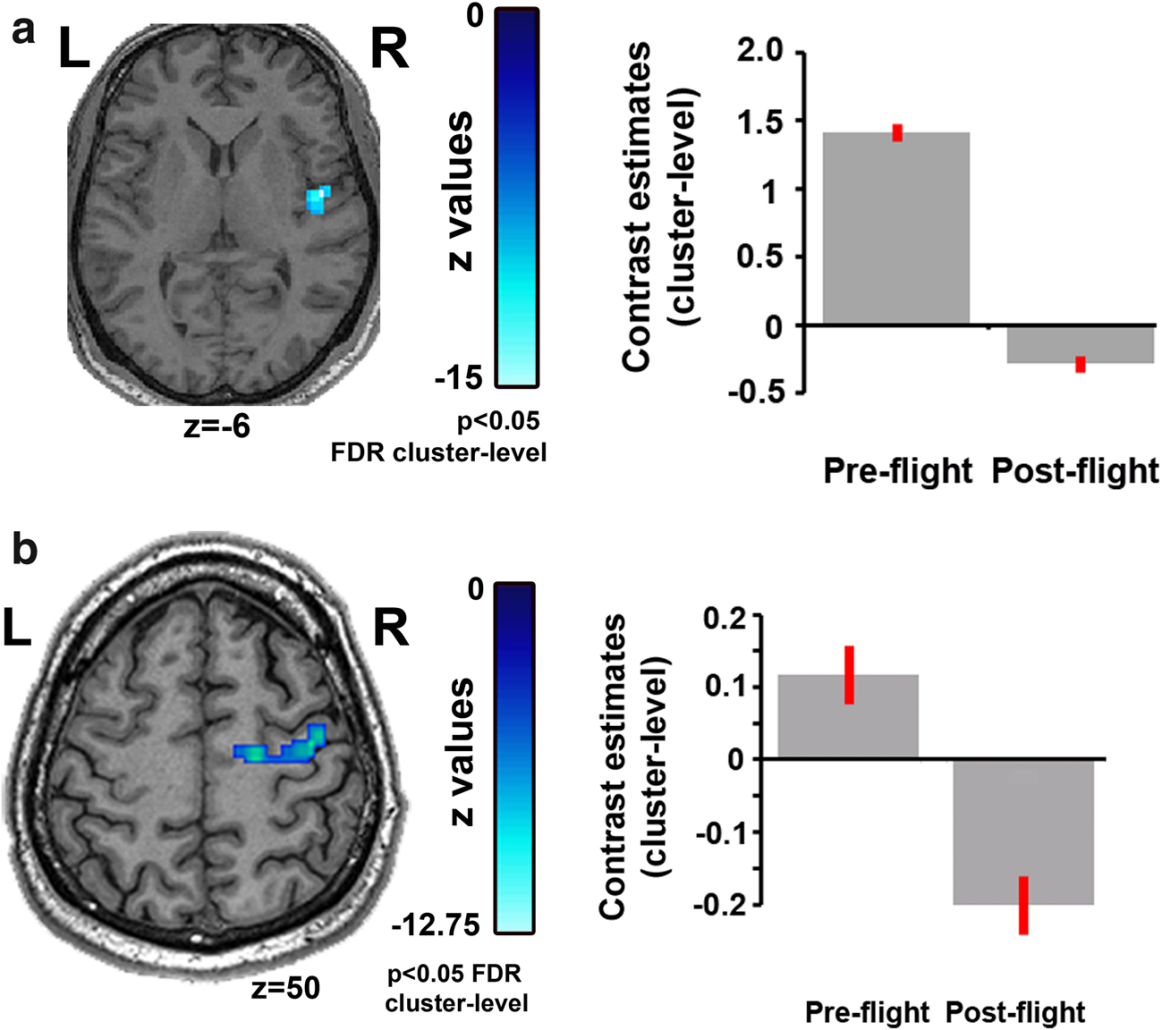
**a** The hypothesis-free exploration of connectivity changes (voxel-to-voxel connectivity analysis) indicated that, compared to pre-flight scan, at post-flight the cosmonaut had reduced intrinsic connectivity in the right insula and ventral posterior cingulate cortex. The figure summarizes the results for the right insula, as this region is a critical part of the vestibular system. **b** The hypothesis-driven network-level estimation of connectivity changes indicated modifications in the default mode network. Secondary seed-by-seed analysis showed that, compared to pre-flight scan, at post-flight the cosmonaut had reduced functional connectivity between the left cerebellum and motor-related regions. *Bars* represent average connectivity strength in the respective cluster with 90 % confidence interval (whiskers) for the pre-flight and post-flight scan. The statistical map is rendered on the normalized MRI scan of the cosmonaut (axial view)

By means of functional MRI investigation of brain activity, we identified altered brain function in the cosmonaut induced by long-duration spaceflight. Specifically, resting state connectivity decreases were identified for the right insula as well as between the left cerebellum and the right motor cortex. The findings that the motor cortex appeared less connected during resting-state and was activated more during the active task could be considered as a compensatory/adaptive response to the microgravity environment as well as to the early post-landing phase. The precentral-, postcentral gyrus and cerebellum, affected in the cosmonaut, are associated with voluntary motor initiation, proprioception, and motor coordination, respectively. Deficits in these brain areas are therefore concomitant with problems of speed and accuracy of aimed movements [primary motor cortex, Brodmann area (BA) 4], somatosensory problems (primary somatosensory cortex, BA3), and movement-timing problems (cerebellum) (De La Torre 2014). Earlier studies on the physiological consequences of spaceflight on motor behavior (Kozlovskaya et al. 1981), e.g., deficits in head-trunk coordination (Tomilovskaya et al. 2013), postural instability (Paloski et al. 1992), and changes in lower limb kinematics with implications on gaze stabilization (McDonald et al. 1996), support the current findings.

We also found a decreased resting state connectivity in the right insula postflight, which is part of the vestibular cortex where the afferents from the otolith organs and semicircular canals converge. This cortical vestibular network is involved in the integration of neurosensory input (i.e., vestibular, visual and proprioceptive input) and its functions include processing of self-motion, spatial orientation, and memory (Brandt et al. 2005), perception of vertical (Lopez et al. 2007) and visual processing related to gravitational cues (Lopez et al. 2009). The reversible problems after spaceflight summed up above have often been attributed to the vestibular system and in particular to the deconditioned gravity sensing otolith system (Kornilova et al. 2012; Moore et al. 2003). The current study, however, suggests that several of these problems originate from alterations at the cortical level, rather than being merely attributed to the peripheral neurosensory organs. Changes in brain function could account for the fact that second time flyers are less prone to some of these problems than first-time flyers, given the process of neural adaptation. However, due to the overall difficulty and restrictions to cosmonaut access, we were only able to conduct the post-flight scan 9 days after return. Theoretically, the observed changes may be attributed not only to long-duration exposure to microgravity but partly also to the re-adaptation to Earth’s gravity. However, the fact that functional connectivity changes were still present 9 days back on Earth shows the importance of the observed findings. This could be relevant in particular for Mars missions, where no on–site ground assistance can be provided and thus, it is important to know the length of the physiological adaptation processes. Only further research can resolve this issue by investigating more space travelers at different intervals post-flight. Additionally, future studies will have to take into account the level of experience, i.e., whether it concerns first-time space travelers or ‘frequent’ flyers.

In conclusion, functional MRI investigation of brain function in a cosmonaut after 6 months exposure to microgravity indicates alterations in vestibular and motor-related regions. These dysfunctions can account for reduced vestibular function and motor control abilities at re-entry. Understanding the effects of spaceflight on the human central nervous system is pivotal for the development of adequate countermeasures. Maximizing crew performance and health is crucial for the success and safety of future prolonged space missions, including missions to Mars. Additionally, our findings may have a clinical relevance, e.g., for vestibular patients who suffer from inadequate neural compensation mechanisms.

*Methods*: Methods and any associated references are available in the online version of the paper.

## Electronic supplementary material

Below is the link to the electronic supplementary material.

Supplementary material 1 (DOCX 53 kb)

Supplementary material 2 (TIFF 6980 kb)
