# Supplementary material for: Cortical reorganization in an astronaut’s brain after long-duration spaceflight
Source: Brain Struct Funct. 2015 May 12;221:2873–6. doi: 10.1007/s00429-015-1054-3 (PMC4884200; doi:10.1007/s00429-015-1054-3)
Supplement: Supplementary file 1 — Supplementary material 1 (DOCX 53 kb) [file 429_2015_1054_MOESM1_ESM.docx]

**Supplementary Online Material**

**Cortical reorganization in an astronaut’s brain after long-duration spaceflight**

Authors: Athena Demertzi*^1^, Angelique Van Ombergen*^2^, Elena S. Tomilovskaya^3^, Ben Jeurissen^4^, Ekaterina Pechenkova^5^, Carol Di Perri^1^, Liudmila D. Litvinova^5^, Enrico Amico^1^, Alena D. Rumshiskaya^5^, Ilya V. Rukavishnikov^3^, Jan Sijbers^4^, Valentin Sinitsyn^5^, Inessa B. Kozlovskaya^3^, Stefan Sunaert^6^, Paul M. Parizel^7^, Paul H. Van de Heyning^2^, Steven S.L. Laureys^1^ & Floris L. Wuyts^2^

* authors have equal contributions

Affiliations:

^1^ Coma Science Group, Cyclotron Research Centre & Neurology department, University of Liège, Liège, Belgium

^2^ Antwerp University Research centre for Equilibrium and Aerospace (AUREA), University of Antwerp, Antwerp, Belgium

^3^ SSC RF – Institute of Biomedical Problems, Russian Academy of Sciences, Moscow, Russia

^4^ iMinds/Vision Lab, University of Antwerp, Antwerp, Belgium

^5^ Radiology Department, Federal Center of Treatment and Rehabilitation, Moscow, Russia

^6^ Department of Imaging & Pathology, Translational MRI, KU Leuven – University of Leuven, Leuven, Belgium

^7^ Radiology Department, Antwerp University Hospital & University of Antwerp, Antwerp, Belgium

**Subjects and procedure**

The cosmonaut and a group of age- and gender-matched healthy controls (n=7, all males, mean age: 37.6y ± 6.46y) were scanned with a 3T MR unit (Discovery 750; GE Healthcare, USA) using a 16-channel head, neck and spine array coil. The data of the healthy volunteers were used to verify whether the extracted intrinsic connectivity networks were accurate in their spatial pattern on the one hand, and to account for the data variance during the statistical comparison between the cosmonaut’s pre- and post-flight scan on the other.

For the resting state condition, subjects were instructed to remain with their eyes closed refraining from any structured thinking, such as counting, singing etc. They simply had to let their minds wander. For the active mental imagery tennis task, before scanning subjects were told that they had to imagine that they stand still at a tennis court, while they use their arm to hit a ball back and forth to an imagined instructor. For the active mental imagery spatial navigation task, subjects were told that they had to imagine that they visit the rooms of their home and to visualize all they would see if they were actually there. When subjects were installed in the scanner, further spoken instructions (“imagine playing tennis”, “imagine visiting the rooms in your home”, “now just relax”) were given at the start of each block. The beginning of each imagery period was cued with the spoken word “tennis” or “navigation,” and rest periods were cued with the word “relax.” Each task was administered as a block design with alternating 30- second periods of task and rest. Each imagery task and the rest condition were repeated 10 times.

**Data acquisition**

During resting state scanning period, 300 multislice T2*-weighted images were acquired with a gradient-echo echo-planar imaging sequence using axial slice orientation and covering the whole brain (voxel size = 3×3×3mm^3^; matrix size = 64×64×42; repetition time = 2000ms; echo time = 30ms; flip angle = 77°; field of view = 192 x 192mm). The same parameters were applied for the mental imagery paradigms (165 acquired volumes per session). For anatomical reference, a high-resolution T1-weighted image was acquired for each subject (T1-weighted 3D magnetization-prepared rapid gradient echo sequence).

**Data analysis**

*Resting state*

The initial three volumes were discarded to avoid T1 saturation effects. Data preprocessing was performed with Statistical Parametric Mapping 8 (SPM8; [www.fil.ion.ucl.ac.uk/spm](http://www.fil.ion.ucl.ac.uk/spm)) and functional connectivity analyses were performed with the CONN functional connectivity toolbox (version 14n) (http://www.nitrc.org/projects/con^n/1^). Preprocessing steps included slice-time correction, realignment, segmentation of structural data, normalization into standard stereotactic Montreal Neurological Institute (MNI) space and spatial smoothing using a Gaussian kernel of 6mm full width at half-maximum (FWHM). As functional connectivity is influenced by head motion in the scanner (van Dijk, Sabuncu, & Buckner, 2012), we accounted for motion artifact detection and rejection using the artifact detection tool (ART; <http://www.nitrc.org/projects/artifact_detect>). Specifically, an image was defined as an outlier (artifact) image if the head displacement in x, y, or z direction was greater than .5 mm from the previous frame, or if the rotational displacement was greater than .02 radians from the previous frame, or if the global mean intensity in the image was greater than 3 SDs from the mean image intensity for the entire resting session. Outliers in the global mean signal intensity and motion were subsequently included as nuisance regressors (i.e. one regressor per outlier within the first-level general linear model). Therefore, the temporal structure of the data was not disrupted. For noise reduction, previous methods subtracted the global signal across the brain, a controversial issue in resting state analyses (Murphy, Birn, Handwerker, Jones, & Bandettini, 2009; Saad et al., 2012; Wong, Olafsson, Tal, & Liu, 2012), and the mean signals from noise regions of interest (ROIs). Here, we used the anatomical component-based noise correction method aCompCor (Behzadi, Restom, Liau, & Liu, 2007), which models the influence of noise as a voxel-specific linear combination of multiple empirically estimated noise sources by deriving principal components from noise ROIs and by including them as nuisance parameters within the general linear models. Specifically, the anatomical image for each participant was segmented into white matter (WM), gray matter, and cerebrospinal fluid (CSF) masks using SPM8. To minimize partial voluming with gray matter, the WM and CSF masks were eroded by one voxel, which resulted in substantially smaller masks than the original segmentations (Chai, Castañán, Öngür, & Whitfield-Gabrieli, 2012). The eroded WM and CSF masks were then used as noise ROIs. Signals from the WM and CSF noise ROIs were extracted from the unsmoothed functional volumes to avoid additional risk of contaminating WM and CSF signals with gray matter signals. A temporal band-pass filter of 0.01- 0.1 Hz was applied on the time series, to restrict the analysis to low frequency fluctuations, which characterize fMRI BOLD resting state activity. Residual head motion parameters (three rotation and three translation parameters, plus another six parameters representing their first-order temporal derivatives) were regressed out.

Statistical analysis adopted a hypothesis-free (voxel-to-voxel) and a hypothesis-driven (seed-to-voxel) approach. First-level voxel-to-voxel analysis encompassed the estimation of voxel-to-voxel functional correlation matrix within each subject. From the residual BOLD time series at every voxel within an a priori gray matter mask (isotropic 2-mm voxels), the matrix of voxel-to-voxel bivariate correlation coefficients was computed (Raichle, 2011). From this voxel-to-voxel correlation matrix, the intrinsic connectivity contrast (Martuzzi et al., 2011) was computed, which characterizes the strength of the global connectivity pattern between each voxel and the rest of the brain. For the first-level seed-to-voxel, multiple seed regions were used to characterize functional connectivity in the default mode network, lateral frontoparietal, salience, sensorimotor, auditory and visual networks. The seeds, which were selected to replicate the networks, were defined as 10mm- (for cortical areas) and 4mm-radius spheres (for subcortical structures) around peak coordinates taken from the literature (mainly from independent component analyses; SOM for coordinates with related literature). For each network, timeseries from the voxels contained in each seed region were averaged together. This averaged timeseries was used to estimate whole-brain correlation r maps, which were then converted to normally, distributed Fisher’s z transformed correlation maps to allow for subsequent group-level analysis.

Following the computation for each subject of voxel-level intrinsic connectivity contrast and seed-to-voxel connectivity maps, these were entered into a second-level general linear model to obtain group-level estimates and inferences. In our analysis, the second-level design matrix included three groups, namely the cosmonaut’s scan at pre-flight, the cosmonaut’s scan at post-flight and the data of healthy controls. With this setup, the necessary variance was obtained in order to perform connectivity analysis with one subject. A t-test between the pre-flight and the second post-flight cosmonaut scan, while modeling the group of controls as zero, were ordered to estimate whether functional connectivity was higher at pre-flight compared to post-flight scan. This contrast was used both for the hypothesis-free and hypothesis-driven analysis. For the network approach, further one-sample t-tests for each network were ordered to estimate network-level functional connectivity for the group of healthy volunteers. False positive control was performed by applying false discovery error rate p< 0.05 at the cluster-level.

*Active mental imagery tasks*

SPM8 was used to identify task-specific activation at the single-subject level. Data were manually reoriented, normalized on the MNI template and smoothed using a 8mm FWHM Gaussian isotropic kernel. Each of the experimental condition was modeled by a boxcar function convolved with a hemodynamic response function with its time derivatives in the GLM. First-level T contrasts for “tennis vs. relax” and “navigation vs. relax” were calculated for each subject. At the second-level analysis, the design matrix modeled the contrast images of the cosmonaut (pre and post-flight) and those of controls. The contrast images were compared for pre and post flight of the cosmonaut, whereas controls’ data were modeled as zero and used only to provide variance to the design matrix. Statistical analyses identified the difference in activation between the pre and post-flight of the cosmonaut. Results are shown at p_uncorrected_<0.05.

**Supplementary Table 1.** Peak coordinates of the regions of interest (ROI) which were selected according to the literature for the replication of the fMRI resting state patterns of the six studies networks. All ROIs were defined as 10mm (for cortical areas) and 4mm-radius (*for subcortical structures) spheres around peak coordinates.

| **Intrinsic Connectivity network** | **Brodmann area**  **[ROI centered at x, y, z]** |
| --- | --- |
| ***Default mode network*** (Raichle, 2011) | |
| Posterior cingulate cortex/precuneus | 31 [0 -52 27] |
| Medial prefrontal cortex | 9 [-1 54 27] |
| Lateral parietal cortex [left] [right] | 39 [-46 -66 30] [49 -63 33] |
| Inferior temporal cortex | 21 [-61 -24 -9] [58 -24 -9] |
| Cerebellum | [-25 -81 -33] [25 -81 -33] |
| Thalamus* | [0 -12 9] |
| Brainstem* (Boveroux et al., 2010) | [12 -24 -24] |
| ***Frontoparietal network*** (Fair et al., 2009) | |
| Dorsolateral prefrontal cortex [left] [right] | 9 [-43 22 34] [43 22 34] |
| Inferior parietal lobule [left] [right] | 40 [-51 -51 36] [51 -47 42] |
| Premotor cortex left [left] [right] | 6 [-41 3 36] [41 3 36] |
| Midcingulate cortex | 23 [0 -29 30] |
| Angular gyrus [left] [right] | 39 [-31 -59 42] [30 -61 39] |
| Precuneus [left] [right] | 7 [-9 -72 37] [10 -69 39] |
| Brainstem* (Boveroux et al., 2010) | [12 -24 -24] |
| Cerebellum (Boveroux et al., 2010) | [-4 -56 -40] |
| Thalamus [left] [right]* (Boveroux et al., 2010) | [-4 -12 0] [4 -12 0] |
| ***Salience*** (Seeley et al., 2007) | |
| Orbital frontoinsula [left] [right] | 12 [-40 18 -12] [42 10 -12] |
| Temporal pole [left] [right] | 38 [-52 16 -14] [52 20 -18] |
| Paracingulate | 32 [0 44 28] |
| Dorsal anterior cingulate [left] [right] | 24 [-6 18 30] [6 22 30] |
| Supplementary motor area [left] [right] | 6 [-4 14 48] [4 14 48] |
| Superior temporal gyrus [left] [right] | 22 [-62 -16 8] [64 -38 6] |
| Parietal operculum [left] [right] | 40 [-60 -40 40] [58 -40 30] |
| Ventrolateral prefrontal cortex | 47 [42 46 0] |
| Dorsolateral prefrontal cortex [left] [right] | 46 [-38 52 10] [30 48 22] |
| Thalamus* | [-12 -18 6] [12 -18 6] |
| Hypothalamus* [left] [right] | [-10 -14 -8] [6 -16 -6] |
| Periaqueductal grey* | [-4 -24 -2] |
| Ventral tegmental area * [left] [right] | [8 -8 -14] [-10 -14 -10] |
| ***Auditory*** (Maudoux et al., 2012) | |
| Superior transverse temporal gyrus [left] [right] | 41/42 [-44 -6 11] [44 -6 11] |
| Precentral gyrus [left] [right] | 6 [-53 -6 8] [58 -6 11] |
| Anterior cingulate cortex | 24 [6 -7 43] |
| Visual cortex | 19 [-6 -88 37] [6 -88 37] |
| ***Sensorimotor*** (Raichle, 2011) | |
| Primary motor cortex | 3 [-39 -26 51] [38 -26 48] |
| Supplementary motor area | [0 -21 48] |
| ***Visual*** | |
| Primary visual cortex (Thomason et al., 2011) | 17 [-13 -85 6] [8 -82 6] |
| Secondary visual cortex (De Luca, Beckmann, De Stefano, Matthews, & Smith, 2006) | 18 [-6 -78 -3] [6 -78 -3] |
| Associative visual cortex (De Luca et al., 2006) | 19 [-30 -89 20] [30 -89 20] |

*4mm-radius sphere ROIs around selected coordinates x,y,z

**Supplementary Figure 1**. The intrinsic connectivity networks as estimated across the group of healthy volunteers (n=7) by means of the seed-to-voxel connectivity analysis. Statistical maps are rendered on a mean normalized T1 (neurological convention) and are thresholded at p<0.05 FDR correction for multiple comparisons, cluster-level. Color bars indicate connectivity strength (T values) and number in parenthesis refer to x y z coordinates.

**References**

Behzadi, Y., Restom, K., Liau, J., & Liu, T. T. (2007). A component based noise correction method (CompCor) for BOLD and perfusion based fMRI. *NeuroImage*, *37*(1), 90–101. doi:10.1016/j.neuroimage.2007.04.042

Boveroux, P., Vanhaudenhuyse, A., Bruno, M.-A., Noirhomme, Q., Lauwick, S., Luxen, A., … Boly, M. (2010). Breakdown of within- and between-network resting state functional magnetic resonance imaging connectivity during propofol-induced loss of consciousness. *Anesthesiology*, *113*(5), 1038–1053. doi:10.1097/ALN.0b013e3181f697f5

Chai, X. J., Castañán, A. N., Öngür, D., & Whitfield-Gabrieli, S. (2012). Anticorrelations in resting state networks without global signal regression. *NeuroImage*, *59*(2), 1420–1428. doi:10.1016/j.neuroimage.2011.08.048

De Luca, M., Beckmann, C., De Stefano, N., Matthews, P., & Smith, S. (2006). fMRI resting state networks define distinct modes of long-distance interactions in the human brain. *NeuroImage*, *29*, 1359–67.

Fair, D. A., Cohen, A. L., Power, J. D., Dosenbach, N. U. F., Church, J. A., Miezin, F. M., … Petersen, S. E. (2009). Functional brain networks develop from a “local to distributed” organization. *PLoS Computational Biology*, *5*(5). doi:10.1371/journal.pcbi.1000381

Martuzzi, R., Ramani, R., Qiu, M., Shen, X., Papademetris, X., & Constable, R. T. (2011). A whole-brain voxel based measure of intrinsic connectivity contrast reveals local changes in tissue connectivity with anesthetic without a priori assumptions on thresholds or regions of interest. *NeuroImage*, *58*(4), 1044–1050. doi:10.1016/j.neuroimage.2011.06.075

Maudoux, A., Lefebvre, P., Cabay, J. E., Demertzi, A., Vanhaudenhuyse, A., Laureys, S., & Soddu, A. (2012). Auditory resting-state network connectivity in tinnitus: A functional MRI study. *PLoS ONE*, *7*(5). doi:10.1371/journal.pone.0036222

Murphy, K., Birn, R. M., Handwerker, D. A., Jones, T. B., & Bandettini, P. A. (2009). The impact of global signal regression on resting state correlations: Are anti-correlated networks introduced? *NeuroImage*, *44*(3), 893–905. doi:10.1016/j.neuroimage.2008.09.036

Raichle, M. (2011). The restless brain. *Brain Connectivity*, *1*, 3–12.

Saad, Z. S., Gotts, S. J., Murphy, K., Chen, G., Jo, H. J., Martin, A., & Cox, R. W. (2012). Trouble at Rest: How Correlation Patterns and Group Differences Become Distorted After Global Signal Regression. *Brain Connectivity*. doi:10.1089/brain.2012.0080

Seeley, W. W., Menon, V., Schatzberg, A. F., Keller, J., Glover, G. H., Kenna, H., … Greicius, M. D. (2007). Dissociable intrinsic connectivity networks for salience processing and executive control. *The Journal of Neuroscience : The Official Journal of the Society for Neuroscience*, *27*(9), 2349–2356. doi:10.1523/JNEUROSCI.5587-06.2007

Thomason, M. E., Dennis, E. L., Joshi, A. A., Joshi, S. H., Dinov, I. D., Chang, C., … Gotlib, I. H. (2011). Resting-state fMRI can reliably map neural networks in children. *NeuroImage*, *55*(1), 165–175. doi:10.1016/j.neuroimage.2010.11.080

Van Dijk, K. R. A., Sabuncu, M. R., & Buckner, R. L. (2012). The influence of head motion on intrinsic functional connectivity MRI. *NeuroImage*, *59*(1), 431–438. doi:10.1016/j.neuroimage.2011.07.044

Wong, C. W., Olafsson, V., Tal, O., & Liu, T. T. (2012). Anti-correlated networks, global signal regression, and the effects of caffeine in resting-state functional MRI. *NeuroImage*, *63*(1), 356–364. doi:10.1016/j.neuroimage.2012.06.035
