# Supplementary figures and images for: Cortical reorganization in an astronaut’s brain after long-duration spaceflight
Source: Brain Struct Funct. 2015 May 12;221:2873–6. doi: 10.1007/s00429-015-1054-3 (PMC4884200; doi:10.1007/s00429-015-1054-3)

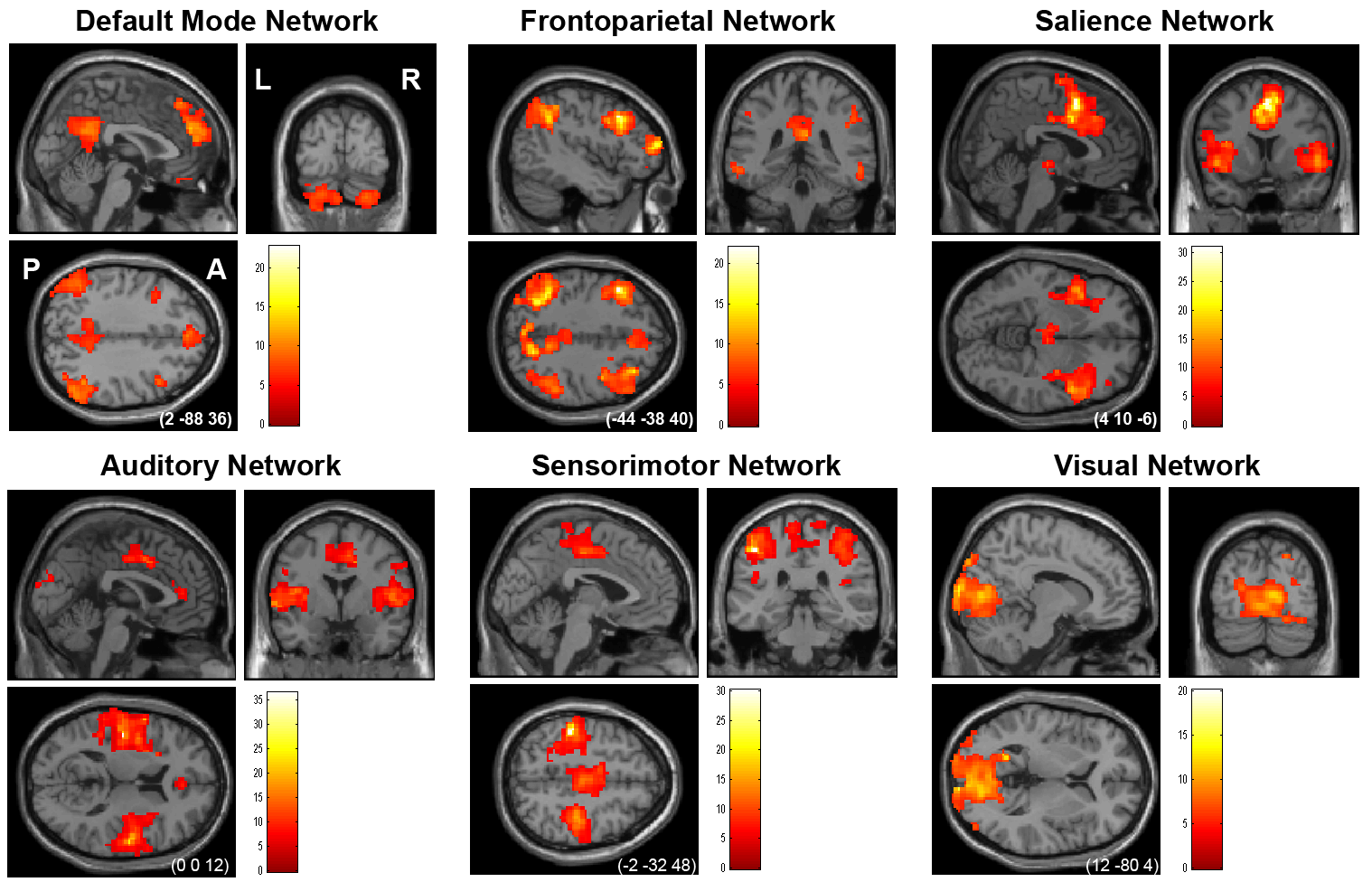

Supplement: Supplementary file 2 — Supplementary material 2 (TIFF 6980 kb) [file 429_2015_1054_MOESM2_ESM.tif]
